# Quantitative proteomics to study carbapenem resistance in *Acinetobacter baumannii*

**DOI:** 10.3389/fmicb.2014.00512

**Published:** 2014-09-26

**Authors:** Vishvanath Tiwari, Monalisa Tiwari

**Affiliations:** Department of Biochemistry, Central University of RajasthanAjmer, India

**Keywords:** quantitative proteomics, *Acinetobacter baumannii*, carbapenem resistance, pathogenesis, biofilm formation

## Abstract

*Acinetobacter baumannii* is an opportunistic pathogen causing pneumonia, respiratory infections and urinary tract infections. The prevalence of this lethal pathogen increases gradually in the clinical setup where it can grow on artificial surfaces, utilize ethanol as a carbon source. Moreover it resists desiccation. Carbapenems, a β-lactam, are the most commonly prescribed drugs against *A. baumannii*. Resistance against carbapenem has emerged in *Acinetobacter baumannii* which can create significant health problems and is responsible for high morbidity and mortality. With the development of quantitative proteomics, a considerable progress has been made in the study of carbapenem resistance of *Acinetobacter baumannii*. Recent updates showed that quantitative proteomics has now emerged as an important tool to understand the carbapenem resistance mechanism in *Acinetobacter baumannii*. Present review also highlights the complementary nature of different quantitative proteomic methods used to study carbapenem resistance and suggests to combine multiple proteomic methods for understanding the response to antibiotics by *Acinetobacter baumannii*.

## Introduction

*Acinetobacter baumannii* is one of the six opportunistic pathogens grouped into ESKAPE pathogens that are linked to the highest degree of mortality as described by Infectious Disease Society of America (Klevens et al., 2006; Boucher et al., 2009). It causes pneumonia, urinary tract infections, respiratory infections and meningitis. *Acinetobacter baumannii* has emerged as a threat to soldiers, wounded during military operations in Iraq and Afghanistan (Davis et al., 2005). The organism can be isolated from natural resources (Kempf et al., 2012). It can grow on artificial surfaces (Espinal et al., 2012) and can utilize ethanol as a carbon source (Navon-Venezia et al., 2005; Fiester and Actis, 2013; Gandhi et al., 2014), resist desiccation, survive at various temperature and pH conditions (Bergogne-Berezin and Towner, 1996), this makes it a lethal pathogen. Prevalence of *Acinetobacter baumannii* in clinical setup has increased gradually (Tiwari et al., 2012a). Commonly prescribed drug against *A. baumannii* are carbapenems which belong to the β-lactam group of antibiotics (Hawkey and Jones, 2009). Resistance against carbapenem has emerged in *Acinetobacter baumannii* which can create significant health problems and responsible for high morbidity and mortality (Sengstock et al., 2010). Reports showed that mortality due to carbapenem-resistant *Acinetobacter baumannii* is about 52% as compared to 19% when infected with carbapenem sensitive variant (Jamulitrat et al., 2009). This makes it one of the major health concerns.

Quantitative proteomics has been employed for both discovery and targeted proteomic analysis to understand global proteomic dynamics of the organism. With the development of quantitative proteomics, a considerable progress has been made in the study of drug resistance. There are various methods which are used in the quantitative proteomics. Oldest method is the comparison of two commassie stained 2D-PAGE gels (Klose, 1975; O'farrell, 1975; Meleady, 2011; Rabilloud and Lelong, 2011). This was followed by emergence of labeled method like Differential In-Gel Electrophoresis, DIGE (Unlu et al., 1997; Yan et al., 2002; Knowles et al., 2003; Timms and Cramer, 2008), Stable Isotope Labeling by Amino acids in Cell culture, SILAC (Ong et al., 2002; Ong and Mann, 2005) Isotope Coded Affinity Tags, ICAT (Gygi et al., 1999; Gygi and Aebersold, 2000), Isobaric tags quantification, iTRAQ (Ross et al., 2004; Desouza et al., 2009) and Isotope-coded protein label, ICPL (Kellermann, 2008; Abdallah et al., 2012; Kellermann and Lottspeich, 2012). Isotope labels can be introduced into peptides metabolically, chemically or enzymatically. Most recently developed methods for quantitative proteomics are label free quantification methods like spectral counting (Mirza and Olivier, 2008; Zhu et al., 2010) and Selected Reaction Monitoring, SRM (Elschenbroich and Kislinger, 2011; Hossain et al., 2011). In label free proteomic methods, mass spectrometer can recognize the mass difference and their quantification is achieved by comparing their respective signal intensities. Proteomic techniques have been used for the identification and quantification of protein samples and its validation have been done by ELISA (Chen et al., 2010), western blotting (Xiaoyu et al., 2013), immunohistochemistry (Perdomo et al., 2012), RT-PCR and real time PCR (Choi and Shim, 2008; Zhang et al., 2011; Paul et al., 2013; Xiaoyu et al., 2013). In the present review, we critically review the use of quantitative proteomics in the study of carbapenem resistance in *Acinetobacter baumannii*. We also discuss the future perspective of the quantitative proteomics in the study of carbapenem resistance as well as limitation of different methods used in quantitative proteomics.

## Quantitative proteomics as a tool to study of carbapenem resistance in *A. baumannii*

Quantitative proteomics has very diverse applications and significance but in the present review, we have explained its significance in the study of carbapenem resistance of *Acinetobacter baumannii*. A number of approaches using quantitative proteomics have been employed for the study of bacterial drug resistance and pathogenesis.

Quantitative outer membrane proteomics between wild type and carbapenem resistance strain of *Acinetobacter baumannii* have been reported (Marti et al., 2006; Siroy et al., 2006; Kwon et al., 2009). Using silver stained 2DE gel, selection of protein spots based on the intensity and sharpness of the spots in the gel; and identification of the protein by homologous matching with other species of *Acinetobacter*, Marti et al., performed an analysis of the major proteins in the membrane fraction of a multidrug resistant strain of *Acinetobacter baumannii* and identify OmpA, ribosomal protein, chaperone and elongation factor (Marti et al., 2006). The result does not highlight the quantitative expression of protein but it signifies the application of the proteomics in the study of carbapenem resistance. Siroy et. al., performed global comparison of the membrane fraction of a sensitive strain with a carbapenem resistant strain of *Acinetobacter baumannii* by comparing two coomassie stained 2D-PAGE gels. Results highlighted that about 36 and 56% protein spots were different in the inner membrane and outer membrane fraction of resistant strain of *Acinetobacter* respectively as compared to the sensitive strain (Siroy et al., 2006). They pointed out that resistance against carbapenem has been developed due to overexpression of RND-type efflux systems and virulence factors like FepA-like and siderophore receptors, absence of PBP1b protein, structural modifications to the CarO porin and presence of different isoforms of the channel OmpW in the carbapenem resistant strain of *Acinetobacter baumannii* (Siroy et al., 2006). This study has given a new direction to the study of carbapenem resistance using quantitative proteomics approach. Similar to approach of Marti et al. (2006), Kwon et al., also used silver stained 2DE gel to identify 132 proteins associated with outer membrane vesicles of *Acinetobacter baumannii* (Kwon et al., 2009). 2D electrophoresis was also used to explain role of different proteins in metabolism using native ATCC strain of *Acinetobacter baumannii* (Soares et al., 2009b). Results highlighted that robust and versatile metabolism of *Acinetobacter* plays very important role in the carbapenem resistance and virulence of *A. baumannii* (Soares et al., 2009b). Using 2DE based membrane proteomic approach; Lee et al., explained the mechanism of hetero-resistance induced by imipenem (a member of carbapenem group) in the multidrug resistant *Acinetobacter baumannii* (Lee et al., 2011). They showed that imipenem treatment leads to the up-regulation of AmpC, Cpn60 chaperonin, ATP synthase, and OmpA (Lee et al., 2011).

Vashist et al., showed the importance of outer membrane in the carbapenem resistance using differential DIGE of outer membrane of carbapenem resistance strain as compared to sensitive strain of *Acinetobacter* (Vashist et al., 2010). They concluded that emergence of carbapenem resistance in *A. baumannii* is due to the decreased expression of CarO, porins (e.g., Omp-A) and increased expression of biofilm forming protein (e.g., CsuA/B) and nutrient transporters (e.g., iron binding protein, ABC transporter). Vashist et al., identified more proteins with significant role in the carbapenem resistance of *Acinetobacter* as compare to Siroy et al. (2006), which further confirm the advantages of DIGE based methods (Vashist et al., 2010) over silver stained methods (Siroy et al., 2006). Similarly, Tiwari et al., identified the importance of the metabolism in the carbapenem resistance of *Acinetobacter* using differential DIGE of inner membrane fraction (Tiwari et al., 2012c). Results concluded that emergence of carbapenem resistance in *A. baumannii* was found to be associated with overproduction of carbapenem hydrolysing β-lactamase (e.g., AmpC and OXA-51) and metabolic enzymes as well as downregulation of surface antigen and OmpW. They also showed that overproduction of these proteins/enzymes have been achieved by enhanced transcription, translation (e.g., Elongation factor Tu and 30S ribosomal protein) and folding (e.g., 60 KDa chaperonin and TCP-1/cpn60 chaperonin protein (Tiwari et al., 2012c).

Yun et al., performed differential quantitative proteomic analysis of cell wall and plasma membrane fractions from multidrug-resistant *Acinetobacter baumannii* using labeled iTRAQ quantitative approach (Yun et al., 2011). They reported that carbapenem also induces the expression of resistance-nodulation-cell division transporters, protein kinases and suppresses outer membrane proteins expression (Yun et al., 2011). This study further advanced the study of carbapenem resistance of *Acinetobacter baumannii* because it used isobaric tag for labeling, reverse phase chromatography for peptide separation and MS/MS mass spectrometry for peptide identification which was relatively more sensitive than older methods. They also compared the result from label as well as label free methods and concluded that more than 80% protein have similar expression (down/up-regulation) pattern for label-free (LC-MS/MS) and labeled (iTRAQ) methods (Yun et al., 2011). This result highlighted the importance to use more than one quantitative proteomic method because of their complementary nature to enhance the reproducibility and validity of quantitative proteomic result. The identified proteins from different quantitative proteomic methods have been listed in the Table 1.

**Table 1:** **Differentially expressed proteins identified in the carbapenem resistance strain as compared to sensitive strain of *A. baumannii* using various quantitative proteomic approaches (Siroy et al., 2006; Kwon et al., 2009; Vashist et al., 2010; Cabral et al., 2011; Lee et al., 2011; McQueary and Actis, 2011; Yun et al., 2011; Tiwari et al., 2012c)**.

**Identified Protein**	**Quantitative Proteomic methods**			**Significance**
	**2DE**	**DIGE**	**Isobaric and other**	
Carbapenem hydrolyzing beta-lactamase (AmpC and OXA)	Yes	Yes	Yes	Hydrolyze beta-lactams and carbapenem
Efflux pumps (AdeABC efflux pump, RND Family transporters)	Yes	Yes	Yes	Aids in the efflux of the antibiotics
Penicillin-binding protein (PBP6, PBP1b)	Yes	No	Yes	Synthesis of peptidoglycan, its alteration cause resistance
Outer membrane protein (OmpA)	No	Yes	Yes	Associated with non-specific transport
CarO protein	Yes	Yes	Yes	Involved in carbapenem resistance
Omp W	Yes	Yes	Yes	Down regulation in resistant strains decreases entry of antibiotics
Biofilm forming protein (Csu A/B)	Yes	Yes	Yes	CsuA/BABCDE chaperone-usher pili assembly system is require for biofilm formation
DcaP like protein	No	Yes	Yes	Associated with cell wall and membrane biogenesis, also have role in biofilm formation
Putative porin, OprD family	No	Yes	Yes	Involved in non-specific transport, also have role in biofilm formation
Signaling protein (tyrosine kinase)	N/D	N/D	Yes	Involved in novel two-component regulatory system which plays role in biofilm formation
Iron-binding protein Receptor (Siderophore receptor)	No	Yes	Yes	Involved in iron transport
Chaperonin	No	Yes	Yes	Aids in stress induced stabilization of protein
Peptidyl-prolyl cis-trans isomerase	N/D	Yes	Yes	Accelerates the folding of proteins by cis-trans isomerization of proline imidic peptide bonds
Regulatory protein (Elongation Factor Tu etc.)	Yes	Yes	Yes	Associated with protein synthesis
Energy producing enzymes (MDH, Aconitate hydratase, ATP synthase etc.)	No	Yes	Yes	Associated with energy production in the cell
Putative tonB-dependent receptor protein	Yes	N/D	Yes	Acts as TonB-dependent receptor for a non-iron nutrient source
ABC transporter	Yes	Yes	Yes	Associated with Phosphate and Amino acid transport
Lipoproteins	Yes	Yes	Yes	Involved in the adhesion and translocation of virulence factors in host cells
Superoxide dismutase	Yes	Yes	Yes	Destroy free radicals produced in the cell
Phosphor-*N*-acetyl muramoylpentapeptide Transferase	N/D	N/D	Yes	Involved in Cell wall synthesis
Cell division protein (zipA)	N/D	N/D	Yes	Involved in cell division
Putative universal stress protein family	N/D	N/D	Yes	Involved in the stress response

*No, No change; Yes, changed in the resistant strain as compared to sensitive strain; N/D, Not detected*.

Pathogenesis of the *Acinetobacter* is also influenced by host-pathogen interaction and nutritional immunity of the host. Role of nutritional immunity in the survival of carbapenem resistance strain of *Acinetobacter* in human host has also been studied using differential proteomic approach (Nwugo et al., 2011; Mortensen and Skaar, 2012; Tiwari, 2013). Nwugo et al., compare the 2DE gel of total lysate and outer membrane fractions isolated from *A. baumannii* sensitive strain cultured under iron-rich and iron-deficient condition. Results indicated that iron-rich condition leads to the overexpression of proteins involved in the iron storage, metabolic process and lipid biosynthesis while iron deficient condition leads to the overexpression of proteins involved in the iron acquisition (Nwugo et al., 2011). Similarly, using DIGE and LC-MS/MS, Tiwari et al., concluded that carbapenem resistant strain of *A. baumannii* upregulates proteins associated with iron acquisition under iron limiting condition while upregulate metabolic enzymes under iron limiting condition (Tiwari, 2013).

High-end isoelectric point (pH 6–11) differential proteome analysis of *Acinetobacter radioresistens* also reveals that envelope stress responses can be induced by aromatic compounds (Mazzoli et al., 2011). Role of proteins detected by the quantitative proteomics methods has been validated by other *in-vitro* or *in-vivo* methods. It was found that *Acinetobacter baumannii* develop resistance against carbapenem via alteration in the expression/activity of β-lactamase (Tiwari et al., 2012a,b,c; Tiwari, 2013, 2014; Tiwari and Moganty, 2013, 2014) and alteration in the penicillin binding protein (Vashist et al., 2011). Upregulation of metabolic enzymes/proteins found in the inner membrane protein also enhanced the carbapenem resistance in *A. baumannii* (Tiwari et al., 2012c).

Biofilm is a functional consortium of microorganisms organized within an extensive exopolymeric matrix (Gurung et al., 2013). Biofilm formation is one of the important causes for the persistence of *Acinetobacter baumannii* on the surface of host epithelial cells and other surfaces (Espinal et al., 2012; Longo et al., 2014). Cabral et al., performed differential proteomics of *Acinetobacter* cultured in three different conditions (exponential, late stationary phase and biofilms stage) using 2D-DIGE and MALDI-TOF/TOF as well as iTRAQ/SCX-LC-MS/MS. They also checked the effects of biofilm inhibitory compound (salicylate) on the biofilm formation. This multiple-approach strategy showed a unique lifestyle of *A. baumannii* involved in biofilm formation (Cabral et al., 2011). CsuA/BABCDE chaperone-usher pili assembly system have been identified in *Acinetobacter* as essential for biofilm formation on plastic (McQueary and Actis, 2011). OmpA and CarO have role in the uptake of amino acid hence also have role in biofilm formation by *Acinetobacter baumannii* (Cabral et al., 2011).

*Acinetobacter baumannii* OMV's induces pathogenesis in the host because of secretion of the outer membrane protein (Kwon et al., 2009) which is cytotoxic to the host (Jin et al., 2011). Secretion of OMVs from *A. baumannii* has been studied using silver stained 2DE based quantitative proteomics approach (Kwon et al., 2009). Soares et al., identified alterations in the plasma proteome of individuals infected with *Acinetobacter baumannii* as compared to healthy controls using DIGE based differential proteomic approach (Soares et al., 2009a).

Using quantitative phosphoproteomics approach, selected phosphorylation sites have been identified in *Acinetobacter* which has been discussed in the context of stress/starvation, pathogenicity and drug resistance (Soares et al., 2014). Qualitative comparison between the Ser/Thr/Tyr phosphoproteomes employed SCX and TiO_2_ chromatography for enrichment of phosphopeptide and LTQ-Orbitrap mass spectrometric analysis for phosphopeptide identification. The percentage distribution of Ser/Thr/Tyr phosphorylation was found to be 68.9% for serine, 24.1% for threonine and 5.2% for tyrosine in sensitive strain as compared to 70.8% for serine, 25.2% for threonine and 3.8% for tyrosine in carbapenem resistant strain of *A. baumannii* (Soares et al., 2014). Phosphoproteomics identified 70 phosphoproteins in the multidrug resistant strain (AbH12O-A2) of *A. baumannii* as compared to 41 phosphoproteins in the sensitive strain (ATCC 17978) of *Acinetobacter* (Soares et al., 2014). Identified phosphoproteins play role in the pathogenesis (e.g., PtK), virulence (e.g., KdpD/KdpE) and drug resistance (Soares et al., 2014). PtK significantly enhances the ability of *Acinetobacter* to grow in human biofluids. KdpD/KdpE, a bacterial two-component signal transduction system, have role in the virulence-related regulatory functions (Soares et al., 2014). This study highlight the significance of high throughput quantitative methods for the study of signaling associated with carbapenem resistance strain of *Acinetobacter baumannii*.

Induction of proteins associated with signaling, putative virulence factors and various stress responses at different stages of *in-vitro* growth has been identified using growth phase-dependent quantitative proteomics using 2-DE and MALDI-TOF/TOF complemented by iTRAQ and LC-MS/MS (Soares et al., 2010; Fiester and Actis, 2013). This result further highlighted the significance of multiple quantitative approaches used together. Extracellular proteome of *Acinetobacter baumannii* has been characterized using 2DE and nanoLC-MS/MS based quantitative proteomic approach (Mendez et al., 2012). Mendez et al., used two protein fractions of the extracellular proteome i.e., outer membrane vesicle (OMV) proteins and freely soluble extracellular proteins (FSEPs) present in the culture medium of *A. baumannii*. The result showed that OMV proteins were found to be associated with pathogenesis and virulence (e.g., CsuE, CsuB, CsuA/B) and secretion systems for delivery of virulence factors while FSEP fraction have extracellular enzymes with degradative activity and role in oxidative stress response (Mendez et al., 2012).

Proteomic experiments also identified differentially expressed lipoproteins as well as proteins responsible for inflammatory/coagulation pathways and kallikrein-kinin system of *Acinetobacter* which will improve future developments in the pathogenesis of the *Acinetobacter* and its therapy (Soares et al., 2009a). In quantitative immunoproteomic approach, potentially immunogenic proteins in *A. baumannii* have been identified using 2DE and MALDI-TOF/TOF mass spectrometric analysis. Immunogenic proteins could serve as antigen for the development of vaccines and passive immunotherapies against *A. baumannii* infections (Bonin et al., 2014).

## Limitation of different quantitative proteomic methods and their significance in the carbapenem resistance studies

There are number of limitations of quantitative proteomic methods that hamper the study of the carbapenem resistance mechanism of *Acinetobacter baumannii*. Merits and demerit of different methods used in the quantitative proteomics have been listed in the Table 2.

**Table 2:** **Different quantitative proteomic approaches with its merits and demerits**.

**Proteomic Tools**	**Merits**	**Demerits**	**References**
**GEL-BASED METHODS**			
2DE	(1) Simplistic	(1) Involve large amount of sample	Klose, 1975; O'farrell, 1975; Meleady, 2011; Rabilloud and Lelong, 2011
	(2) Robust	(2) Low throughput	
	(3) Suitable for MS analysis	(3) Poor recovery of hydrophobic proteins	
		(4) High inter-gel variability	
2D-DIGE	(1) Multiplexing	(1) Expensive Cy dyes	Unlu et al., 1997; Yan et al., 2002; Knowles et al., 2003; Timms and Cramer, 2008; Chen et al., 2010
	(2) Better quantitation	(2) Poor recovery of hydrophobic proteins	
	(3) Minimized gel to gel variation	(3) Difficulty in separation of low molecular weight	
**GEL-FREE METHODS**			
SILAC	(1) High throughput	(1) Suitable only for tissue culture models	Ong et al., 2002; Ong and Mann, 2005; Elliott et al., 2009
	(2) Robust	(2) Costly reagents	
	(3) Sensitive and simple	(3) Not applicable to tissue sample	
ICAT	(1) Selectively isolates peptide	(1) Cannot identify proteins with less than 8 cysteines	Gygi et al., 1999; Gygi and Aebersold, 2000; Toyo'oka, 2012
	(2) Compatible with any amount of protein	(2) Size of ICAT label is large (≈500Da)	
	(3) Complexity of the peptide mixture is reduced	(3) Post-translational modification information is frequently lost	
iTRAQ	(1) Applicable to versatile samples	(1) Involve high amount of sample	Ross et al., 2004; Desouza et al., 2009
	(2) Multiplexing	(2) Incomplete labeling	
	(3) Better quantitation	(3) Expensive reagents	
ICPL	(1) High-throughput quantitative proteome profiling on a global scale	(1) Isotopic effect of deuterated tags that interferes with retention time of the labeled peptides during LC	Kellermann, 2008; Abdallah et al., 2012; Kellermann and Lottspeich, 2012
	(2) Able to detect post-translational modifications and protein isoforms		
	(3) Applicable to protein sample like extracts from tissues or body fluids		
Label-free	(1) Involve less amount of sample	(1) High throughput instrumentation	Mirza and Olivier, 2008; Zhu et al., 2010
	(2) Higher proteome coverage	(2) Not suitable for low abundant proteins	
	(3) Avoid labeling	(3) Incomplete digestion may introduce error	
		(4) Multiplexed analysis is not possible in one experiment	
SRM	(1) Highly sensitive, quantitatively accurate and highly reproducible	(1) Limited broad scale application because of difficulty of generating high-quality SRM assay.	Elschenbroich and Kislinger, 2011; Hossain et al., 2011
	(2) Protein detection is relatively rapid and straightforward	(2) Sensitivity is not comparable to immunological assays	
	(3) Enable the detection of low (>10 ng/ml) abundance proteins	(3) Detection and quantification of low abundance proteins (i.e., ~ 10 ng/ml or less)		
	(4) Quantification of post-translational modification		

Table 2 showed that most of the methods used in quantitative proteomics did not provide information about post-translational modification and they are unable to detect the small proteins. These two limitations can cost heavily in the studies of carbapenem resistance. Quantitative proteomics is unable to produce all information required to confirm role of protein/biomolecule in carbapenem resistance but it gives global insights about resistance mechanism of *Acinetobacter baumannii* which helps in the subsequent studies. Quantitative proteomic methods are also unable to give direct confirmation of the role of protein in the carbapenem resistance. Similarly the result of quantitative proteomics is also influenced by protein extraction procedure. These limitations conclude that quantitative proteomics help to a large extent in the study of carbapenem resistance but it requires help of other methods for confirmation. Therefore, we can say that emergence of quantitative proteomics is really an added advantage in the study of carbapenem resistance.

## Future perspectives of quantitative proteomics to understand carbapenem resistance

Fluorescence based DIGE methods and isobaric based iTRAQ methods in combination with LC-MS/MS are more popular approach used to study carbapenem resistance mechanism of *A. baumannii*. Phosphoproteomics and immunoproteomics have also emerged to understand virulence and pathogenesis of carbapenem resistant strain of *A. baumannii*. Every proteomic method has its own merits and demerits (Table 2) but they provide complementary information. Therefore, combining multiple methods together is an added advantage. This approach has been recently used by some groups to produce valid results (Cabral et al., 2011; Yun et al., 2011). Literature based on proteomic methods has showed the presence of differentially expressed proteins in the carbapenem resistant strain but their actual role in carbapenem resistance has not been confirmed using knockout or auxotroph for resistance factors. Therefore, quantitative proteomic methods also require support from non-proteomic methods. With the development of the quantitative immunoproteomics, phosphoproteomics and other omics methods, the identification of the differentially expressed resistance factors become more reliable. Emergence of quantitative proteomic methods like ICPL and SRM methods will help to remove the shortcoming of the routinely used present methods. Future of quantitative proteomics will depend on the use of two quantitative proteomic methods together with better validation methods to outcome the limitation of the current methods or approach.

## Conclusions

The quantitative proteomics has overcome some of the limitations of other approaches for investigating carbapenem resistance. With the development in the quantitative proteomics, new opportunities are now open to study difficult and challenging tasks. Because of complementary nature of different quantitative proteomic methods, it has been suggested to combine multiple quantitative proteomic methods for the better understanding of carbapenem resistance in *Acinetobacter baumannii*.

### Conflict of interest statement

The authors declare that the research was conducted in the absence of any commercial or financial relationships that could be construed as a potential conflict of interest.
